# What we have learned from the Methadone Maintenance Treatment of Dual Disorder Heroin Use Disorder patients

**DOI:** 10.3390/ijerph16030447

**Published:** 2019-02-03

**Authors:** Angelo G.I. Maremmani, Matteo Pacini, Icro Maremmani

**Affiliations:** 1 Department of Psychiatry, North-Western Tuscany Region NHS Local Health Unit, Versilia Zone, 55049 Viareggio, Italy; angelo.maremmani@uslnordovest.toscana.it; 2 Association for the Application of Neuroscientific Knowledge to Social Aims (AU-CNS), Pietrasanta, 55045 Lucca, Italy; 3 G. De Lisio, Institute of Behavioral Sciences, 56127 Pisa, Italy; paciland@virgilio.it; 4 V.P. Dole Dual Disorder Unit, 2nd Psychiatric Unit, Santa Chiara University Hospital, University of Pisa, 56126 Pisa, Italy

**Keywords:** Dual Disorder, Heroin Use Disorder, Mood Disorders, Anxiety Disorders, Psychotic Disorders, Violence, Alcohol Use Disorder, Methadone Maintenance Treatment

## Abstract

Mental Disorders and Heroin Use Disorder (HUD) often co-occur and constitute correlated risk factors that the authors believe are best considered from a unitary perspective. In this article we review and discuss data collected by the V.P. Dole Research Group in Dual Disorder (V.P. Dole DD-RG) patients according to the following six discussion points: (1) Treatment of personality disorders during Methadone Maintenance Treatment (MMT); (2) Treatment of Mood Disorders during MMT; (3) Treatment of Anxiety Disorders during MMT; (4) Treatment of Psychotic Disorders during MMT; (5) Treatment of violence during MMT; (6) Treatment of Alcohol Use Disorder (AUD) during MMT. In treating Mood Disorder in HUD patients, we must bear in mind the interactions (potentiation and side effects) between psychopharmacology, used substances and agonist opioid medications; the use of psychiatric medications as an anti-craving drug, and the possible use of agonist and antagonist opioid medications in treating the other mental disorders. In treating chronic psychosis in HUD patients, we must consider the potentiation and side effects of antipsychotic drugs consequent on HUD treatment, worsening addiction hypophoria and inducing a more severe reward deficiency syndrome (RDS) in hypophoric patients. Violence and AUD during MMT can benefit from adequate dosages of methadone and co-medication with Sodium gamma-hydroxybutyrate (GHB). The experience of our V.P. Dole DD-RG suggests the following: (a) DD is the new paradigm in neuroscience in deepening our understanding of mental health; (b) To successfully treat DD patients a double competence is needed; (c) In managing DD patients priority must be given to Substance Use Disorder (SUD) treatment (stabilizing patients); (d) Antidepressant use is ancillary to SUD treatment; antipsychotic use must be restricted to acute phases; mood stabilizers must be preferred; any use of Benzodiazepines (BDZs) must be avoided.

## 1. Introduction

The first step in structuring Dual Disorder (DD) effective treatment is the definition of psychiatric diagnosis; this is not easy, because there is an overlap area between outbursts of primary mental disorders and drug- or alcohol-related psychopathology.

Psychiatric illness and Substance Use Disorders (SUD) share several features: Substance use may elicit or else mask a concurrent, but independent psychiatric symptomatology. In this case, it is difficult to differentiate between them. The clinical severity, duration, and typology of mental features have been shown to be linked to the quantity and duration of underlying SUD. Alcohol and other drugs may bring forward the onset of mental disorders for which an autonomous proneness already exists, can exacerbate symptoms of current psychopathology or favor relapses into major syndromes. Conversely, mentally ill individuals may resort to substance use to soothe psychiatric symptoms or to counter the side-effects of various administered drugs. Substance withdrawal can be another cause of psychopathology. SUD may also coexist side by side with independent psychiatric diseases. Lastly, there is extensive overlap between the behaviors that accompany some types of mental disorder and drug-related behaviors [1]. 

When two independent medical disorders are present in the same subject, we use the term ‘Dual Disorder’. In the fields of psychiatry and SUD, the term has taken on the meaning of “the coexistence of a psychiatric disorder with a SUD”. In this article, we will use the acronym ‘DD’ to indicate a dual disorder.

## 2. Treatment of Personality Disorders during Methadone Maintenance Treatment (MMT)

Treece and Nicholson first verified that some personality features (according to the Diagnostic and Statistical Manual of Mental Disorders (DSM) [2] indicate a need for higher methadone stabilization dosages, whereas others tend to lower methadone dosages. Methadone-treated patients and street SUD patients were divided into three groups, according to their personality disorder (PD), plus a fourth category for Heroin Use Disorders (HUD) with a non-pathological personality. Street HUD had been made accessible by utilizing a newspaper advertisement. The A-cluster included schizoid, schizotypal and paranoid personalities characterized by oddity, loneliness, and isolation. The B-cluster comprised narcissistic, borderline, histrionic and antisocial personalities, which were regarded as displaying dramatic, overemotional, eccentric styles. Antisocial personality disorder was shown by 75% of the subjects. The dosages of methadone turned out to be higher in the A- and B-cluster groups than in the non-pathological group [3].

The following is an example of PD and HUD: It was that of a 29-year-old white male addict, of middle-class origins, suffering from chronic depression and schizotypal PD, and treated with 100 mg/day of methadone. When 18, he started displaying isolation, depressive features and antisocial behavior. He first used narcotics during his military service. He also used marijuana, amphetamines, hallucinogenic drugs, and barbiturates occasionally, but in high amounts. His heroin-use quickly became massive and regular. He started MMT at 23, after four unsuccessful detoxification programs, but continued to use alcohol and benzodiazepines (BDZs) even after 14 months of treatment, while displaying flattening of emotions, low self-esteem, and stereotypical speech, lateness, repetitiveness, thought inconsistency, and lying [3].

Our group verified that methadone dosages depend on the grade of psychopathology and aggressiveness at treatment entrance [4]. A sample of 20 subjects was divided into two sub-groups according to the baseline mean SCL90-score (high psychopathological symptomatology vs. low psychopathological symptomatology). All patients had been abstinent from all substances and had achieved a satisfactory psychosocial adaptation, after a treatment period of variable length (1–96 months). Stabilization dosages were 39 ± 23 mg/day (range of 7–80). A higher degree of psychopathology correlated with higher stabilization dosages (60 mg/day vs. 30 mg/day, the latter corresponding to a lower degree of psychopathology); similarly, more top violence accounted for higher stabilization dosages (50 mg/day vs. 30 mg/day for mildly aggressive subjects). Neither violence nor psychopathology appeared to vary with treatment duration. Methadone-sensitive psychopathology appeared to comprise paranoia, depression, somatic symptoms, phobic anxiety, and psychotic symptoms, the latter two showing the strongest correlations. As regards violence, methadone dosage seemed to be related to unexpressed aggression, irritability and violence, the most active associations occurring for the latter two. In summary, the higher the level of psychopathology and hostility at treatment entrance, the higher the methadone dosage required for stabilization.

## 3. Treatment of Mood Disorders during MMT

### 3.1. Heroin Use Disorder and its Consequences on Mood

Opiates usually generate mood disorders during intoxication, while chronic opiate use induces a fall in Central Nervous System noradrenergic firing. Unlike other used substances, opioids are unlikely to cause psychotic syndromes. During manic episodes, substance use may depend on loss of inhibition, impairment of judgment, impulsiveness, or lack of care. SUD patients with mixed episodes are twice as likely to use substances than healthy subjects. The switching phase can be actively unpleasant and lead to substance use as self-medication.

In contrast, other authors judge that mood lability develops as a consequence of Central Nervous System (CNS) neuroadaptation to chronic exposure to heroin. The primary hypothesis is that heroin-induced depression stems from functional alterations in the endorphinergic, noradrenergic and hypophysis-adrenal gland system. The protracted use of heroin adaptation may continue for several months after detoxification and come to cause what is clinically described as hypophoria [5]. Detoxified HUD patients generally show a “protracted withdrawal syndrome”, or a “post-withdrawal syndrome”, which features chronic residual and often disabling withdrawal symptoms [6,7,8,9,10]. The clinical picture points to an organic mood syndrome, that methadone can resolve, and represents the crucial risk factor for relapse into heroin use, if methadone is not at disposal. Dysphoria is usually linked with an increase in craving and substance-seeking activity. Relapse into heroin use followed by a lessening of dysphoria works to refuel the vicious addiction circle, even when other features of early or protracted withdrawal are not present. Mood disorders also arise during detoxification from an opiate. Depression seems to occur more frequently among HUD patients who have gone through methadone tapering (60%) than among those applying for MMT after heroin cessation (25%) [11]. We must consider that HUD patients with mood disorders tend to enter an MMT program, as this is the only treatment capable of restoring the heroin-related opioid imbalance and its associated psychopathology. It is probable that mood alterations, which led subjects to undertake MMT in the first place, will re-emerge after therapeutic stabilization has been achieved.

### 3.2. Treatment of Mood Disorders in HUD Patients

Reducing opiate use may itself induce the onset of psychiatric disorders (psychosis, mania, depression) that predispose the subject to the risk of relapsing into heroin use. When mood disorders are unrelated to SUD, doctors should be careful about using agents associated with use liability. They must also take into account the possible interactions with other psychotropics (e.g., BDZs). MAOIs (Monoamine Oxidase Inhibitors) should be avoided, to prevent interactions with cocaine, heroin or other psychotropic drugs [12,13,14]. In general, fast-acting BDZs (diazepam, alprazolam) should be avoided too, because they have a high addictive potential. Patients who are compliant with the proposed treatment, and who continue under medical supervision, can be safely treated with slow-acting BDZs (oxazepam, clorazepate), which in these cases are safe and appear to reduce the use of fast-acting BDZs. Any other psychotropic drug should be evaluated by urinalysis. In MMT-HUD-and BDZ-misusing patients, a long-lasting, potent and slow-acting benzodiazepine (BDZ), such as clonazepam, which is therefore free of addictive properties, can be used as a replacement for other BDZ compounds [15,16].

Codependence with alcohol, cocaine or other substances is a frequent complication of HUD. 60% of MMT patients entering treatment were abusing cocaine at the time. Cocaine use is found in about 40% of HUD patients, BDZ use is quite common, and alcohol use is problematic in 15–30% of cases [17,18,19,20]. No similar data on naltrexone-maintained patients are available. Even so, it does seem that polyuse is frequent among patients entering a clinically inappropriate naltrexone treatment; they, in fact, refuse or are denied better-fitting options, due to MMT stigma or environmental pressure, together with and cultural bias [21].

Particular caution is required when treating SUD patients suffering from additional psychiatric disorders, as the intervention on HUD alone, even when successful, cannot be expected to resolve the problem of polyuse. Such patients must be carefully monitored (daily alcohol test, twice-a-week urinalyses), with specific pharmacotherapy (e.g., disulfiram), more frequent counselling sessions, and direct access to self-help groups (e.g., Alcoholics Anonymous) and Agonist Opioid Treatment (AOT) programs [22].

Few studies [23,24] have shown that high dose MMT, when combined with many ancillary facilities, can decrease cocaine use. In general, polyuser HUD patients, particularly those taking CNS depressants, should be stabilized on MMT and gradually detoxified from other substances. Treating all different kinds of use at once is bound to fail [22]. We recommend that use issues should be faced one by one.

#### 3.2.1. Antidepressants

Despite the frequency of depressive disorders among HUD patients, as documented in the literature, few reports are available on the use of tricyclic antidepressants. Doxepin administered at doses ranging between 25 and 150 mg once a day in the evening, can improve the data on anxiety, depressive features and anxiety-related insomnia symptomatology [25]. Amitriptyline partly limits withdrawal symptoms in abstaining volunteers [25]. In a double-blind, placebo-controlled study of doxepin in depressed SUD patients, a significant improvement was shown by the Zung and Beck Hamilton rating scales. Although many probands dropped out, retained subjects showed a decrease in craving [26]. Later studies performed on methadone-treated patients failed to show any more significant improvement in imipramine-treated patients with doses ranging between 150 and 225 mg/day vs. placebo. In this case, a general decrease in depressive symptoms was noted [13].

Thus, MMT accounts for the improvement of depressive symptoms, no further advantage being provided by imipramine. In cases of severe depression, the parenteral intake of 25-50 mg of clomipramine ensures rapid and significant improvement, and impressive results after just one week [27]. An ongoing decrease in severity marks the natural course of depressive symptoms after starting MMT through the first eight months [28,29,30,31,32]. Tricyclic agents should, therefore, be resorted to only when symptomatology shows no significant improvement during MMT, and when, consequently, the risk of relapse is high [28,29,30,31,32]. Caution is also needed in light of several cases of tricyclic use that have been documented in the literature [33,34]. According to our V.P. Dole Dual Disorder Research Group (V.P. Dole DD-RG), doses of 150 mg/day are useful in treating most of the cases of depression in HUD patients. Tricyclics, dopaminergic agents like bupropion and trazodone, are helpful during methadone tapering, at the end of a successful program, or, in the first six months after the successful accomplishment of a program, endeavoring to favor abstinence in drug-free subjects, due to their property of limiting mild withdrawal symptoms (protracted withdrawal states, or enduring insomnia).

In conclusion, clinical trials on the effectiveness of tricyclic antidepressants have given ambiguous results. This fact may be partly attributed to the difficulty of retaining abstaining HUD patients in any unspecific treatment. To summarize, it may be said that trials on doxepin have shown its efficacy in methadone-maintained subjects, at doses ranging between 25 and 100 mg. Otherwise, no significant effect has been ascertained either for imipramine or desipramine. However, desipramine blood levels are higher than expected in methadone-maintained subjects [35].

As regards Serotonergic System Reuptake Inhibitors (SSRIs), their effectiveness and safety have been documented by our V.P. Dole DD-RG on subjects displaying recurrent depression by maintaining at average methadone doses of 100 mg/day. We must bear in mind, though, that SSRI bioavailability rises in methadone-maintained patients. Both fluoxetine and fluvoxamine may increase methadone blood levels significantly (by up to 200%, in the case of fluvoxamine) [36]. During the first two weeks of administration, sertraline increases methadone blood levels [37]. Methadone doses should, therefore, be used carefully, when SSRIs are added on in the induction phase. Remarkably, fluvoxamine has proved useful in improving the bioavailability of methadone over 24 hours, in high dose-treated patients, who report withdrawal symptomatology before each new administration (probably due to a fast metabolism). Patients who show an unsatisfactory response to methadone at 100–150 mg/day can benefit from the addition of fluvoxamine [38,39].

MAOIs’ stimulating properties, which have been documented in depressed non-SUD patients too, make them unfit for use with HUD patients, due to their proneness to misuse. Moreover, the likelihood of “Swiss-Cheese effect accidents is too high in HUD patients, who are known to have no control over their consumption of food, chemicals, or alcohol. In prognostic terms, the presence of mood symptoms predicts poorer control over addictive forms of conducts, heavier psychosocial impairment, and greater suicidal risk.

#### 3.2.2. Mood-Stabilizing Medications

Bipolar syndromes are probably the psychiatric disorders best represented among HUD patients. As described in one of our articles, 39 out of 40 our consecutive HUD Methadone Maintained patients received a diagnosis of bipolar I or bipolar II disorder or displayed hyperthymic temperament, or else had a family history of bipolarity [40]. The use of mood stabilizers is correct in patients with DD bipolar disorders or borderline personality disorder [41,42], which are both categories that often involve SUD. However, lithium and carbamazepine have not been clearly shown to be suitable for bipolar HUD patients [37,43]. Moreover, we must bear in mind that the normalization of basal mood does not ensure control over heroin relapse, once the revolving door phase has been entered. Mood stabilization may be decisive in avoiding relapses in the honeymoon phase, or in subjects who can stay persistently abstinent after detoxification. DD/HUD bipolar patients have poorer results than their non-abusing peers. The response to lithium of these subjects is predictably poor, whereas better results can be expected if anticonvulsants, especially valproate, are used. However, lithium may reasonably be used in bipolar cocaine SUD patients [44,45,46].

Lithium-methadone interactions have been suggested on an experimental basis, but has not yet been clinically confirmed [47,48]. Carbamazepine, phenytoin, and phenobarbital strongly decrease the bioavailability of methadone, so precipitating opiate withdrawal [37]. Valproic acid and phenobarbital do not seem to have this effect.

#### 3.2.3. Opioidergic Agents

**Agonists.** Properties typical of antidepressant medications have been discovered for opioids, so suggesting that opioid use may start as a form of self-medication for depressive symptoms. This fact supports the endorphinergic hypothesis for dysthymic disorders. The administration of opioid medications to depressed patients has shown some efficacy, but failures have been reported too. Beta-endorphins, in two trials, were successful in treating depression in a few non-addicted depressed patients (there were two responders in one trial and 3—out of 6—in another) [49,50]. Compared with placebo, the efficacy of beta-endorphins was confirmed, but no greater efficacy over placebo was documented for morphine or methadone, on in non-dependent depressed patients [51]. In HUD patients, higher methadone doses (over 100 mg/day) are needed to stabilize depressed and violent patients at program entrance [4]. In a two-year follow-up, MMT seemed successful in achieving major mood stabilization in bipolar 1 patients [52]. These data have been confirmed in an eight year follow-up [53]. Though contrasting data do exist [54,55], some neurobiological observations are consistent with that orientation. In hypothalamic and limbic areas there is a high concentration of opioid receptors and endorphins, both involved in the physiology of mood disorders; and opioid systems have been shown to interact with catecholaminergic systems, which are involved in the depression pathophysiology. This fact is in agreement with Extein’s hypothesis that “a decrease in endorphinergic activity may be the pathophysiological basis of depression” [56].

It is possible to combine standard stabilization dosages of methadone with standard doses of acid valproic, clozapine, olanzapine, quetiapine, clomipramine, trimipramine and citalopram. As recorded in the experience of our V.P. Dole DD-RG, we used caution during the methadone induction phase of AOT, and we re-evaluated methadone dosage by introducing carbamazepine, fluoxetine, fluvoxamine, sertraline, haloperidol and risperidone, once patients were already in treatment [57].

**Antagonists.** Opiates are known to develop euphoric states, and natural states of elation are associated with high CNS levels of endorphins, but a low frequency of manic states has been reported among HUD patients. The opiate antagonist naloxone, which has no apparent effect on depressed patients, has proved to have antimanic properties [58]. One hypothesis, resulting from observations on SUD or non-SUD subjects, is that naltrexone has a negative influence on basal mood, by observations on SUD or non-SUD subjects. One patient in treatment with naltrexone for its bulimia developed panic attacks [59]. 13 out of 80 naltrexone-maintained patients who were also receiving psychosocial treatment, experienced an overdose during the first year of treatment. Four of these overdoses were lethal overdoses, including one case of suicide. Attempted suicides were found in four of nine non-lethal overdose cases [60]. Data gathered by our V.P. Dole DD-RG indicate that naltrexone treatment is less effective on the aggressive behavior and suicidal thoughts of HUD patients [21,61,62]. This problem emerges most clearly in long-term treatment programs [62]. By contrast, bipolar patients with a low opiate craving can benefit from naltrexone maintenance. They show a satisfactory retention rate compared with uncomplicated SUD patients or non-bipolar SUD patients. The use of fluoxetine as the add-on to naltrexone maintenance has been shown to improve patients’ outcomes, suggesting that naltrexone has an anti-reward property, that is specifically reversible through fluoxetine’s antidepressant effects [63,64].

### 3.3. Proposals

We propose the following strategies when treating DD patients with affective disorders:

- Antidepressant pharmacotherapy alone cannot extinguish addictive behavior in HUD patients.
- Long-acting opiates have antidepressant properties.
- Over-standard dosages of methadone (up 120 mg/day) are needed.
- Antidepressant medications (especially SSRIs) increase blood levels of methadone.
- SSRIs must be used in rapid metabolizer patients on methadone.
- Caution is needed during the MM induction phase.
- SSRIs are not useful during the detoxification of patients with HUD.
- Craving increases during manic phases. Switching antidepressants must be avoided. Anti-craving antidepressants (fluoxetine or sertraline) in depressed HUD patients must be preferred.
- Avoid the Inhibitors of the Monoamine Oxidase, because of their interaction with cocaine (disulfiram effect).
- BDZ for treating comorbid anxiety must be avoided (long-acting opiates have anxiolytic properties).
- The co-use of clomipramine and methadone can reduce the latency of the antidepressant effect.
- Tricyclic antidepressants, bupropion and trazodone, can be used after opioid detoxification for at least six months as anti-hypophoric agents.
- In HUD patients it is possible to find tricyclic use (especially amitriptyline) and tricyclic withdrawal syndrome.
- Mood stabilizers are useful in Bipolar HUD patients, but mood stabilizing therapy alone does not extinguish addictive behavior in HUD patients.
- Caution with carbamazepine is needed. Methadone dosage must be increased if carbamazepine is necessary. This increase is not necessary with valproic acid. Lithium can be used only in compliant cocaine user HUD patients.

## 4. Treatment of Anxiety Disorders during Methadone Maintenance Treatment

Symptoms of anxiety can be found in the addictive history of many SUD patients [65,66,67,68,69,70,71,72]. In Alcohol Use Disorder (AUD) patients, as much as 50–70% of that symptomatology can be described as phobic syndromes, generalized anxiety and panic disorder. Anxiety features, which are found at an overall frequency of around 80%, are even more common among specific groups of cases during withdrawal or intoxication states, where that frequency rises. Many authors have indicated a genetic link between anxiety and addictive disorders and that link has been interpreted as depending on a self-medicating dynamic [73]. Although it is hard to decide whether anxiety is of a primary type or is secondary to SUD, it can be agreed that comorbid anxiety disorders in AUD or HUD patients deserve specific clinical attention and therapeutic intervention.

Any of the DSM-5 anxiety disorders can become manifest during intoxication or withdrawal, no matter what substance is used. The feature typical of panic disorders, phobias, and generalized anxiety are prominent in the most common pictures. DSM-5 indicates syndromes, such as substance-induced anxiety disorders and states that prominent symptoms comprise obsessions and compulsions, free anxiety and panic attacks. The onset of symptoms can start less than one month after intoxication or withdrawal states and those symptoms may continue for months, causing significant psychosocial and working impairment, as well as difficulties in managing private life. Comorbid anxiety disorders can sometimes be classified as a real DD, but their features are not distinguishable from drug-induced ones. Despite this, DSM-5 provides useful criteria for drawing a distinction: When anxiety symptoms forerun the onset of substance use the likelihood of an anxiety disorder being primary rises; when symptoms continue far beyond an episode of intoxication/withdrawal; the same is true when they exceed what might be expected from the severity of the substance intake. Lastly, an anxiety disorder history unrelated to any SUD condition makes a diagnosis of primary anxiety disorder more likely.

Apart from conditions of intoxication/withdrawal, the treatment of anxiety in addicted patients does not differ from the medical treatment of anxiety syndromes. Anti-anxiety agents are indicated for patients who continue to display anxiety even when receiving adequate treatment for their SUD. Target symptoms should always be defined and monitored, and treatment should not necessarily be thought of as chronic. This fact is particularly true of BDZs, which are useful only to the extent to which they prompt patients’ compliance with other treatments. Agents, such as diazepam, lorazepam and alprazolam should be avoided, because of their strong use of liability. In HUD patients, diazepam is one of the most popular psychotropics to be used, not because of its property of alleviating some of the opiate withdrawal symptoms. As HUD patients themselves report, it is often used to maintain euphoria, or to reproduce a heroin-like euphoria when methadone is being taken [74], if heroin itself produces few strong sensations, or else to make a subject feel “high” [26,75]. Clonazepam, on the other hand, in dosages of up to 0.50 mg three times per day, has, when required, proved to be safer and more suitable and is a good alternative. These findings were replicated from animal studies, in which diazepam has been shown to increase the effects of opiates [76]. Diazepam, at high doses, is mostly used to counteract withdrawal symptoms or to mitigate the results of rapid detoxification, or to prolong abstinence after rehab has been completed.

During MMT too, diazepam use is a common finding, more so than among AUD patients [13,25,26,74,75,77,78,79]. The percentage of MMT subjects using BDZs is around 10–20%, reaching a maximum of 30%, if hypnotics or BDZ have been used during the previous week [34,77,80]. According to the Treatment Outcome Prospective Study, between 5% and around 15% of MMT subjects have been using BDZs weekly or less often [81]. As assessed by random urinalysis, regular diazepam use is common too, and 20% of patients are high-rate diazepam users (with more than three substances present in urinalysis results over six months), and around 45% were defined as low-rate users (with at most one result) [82]. Whether BDZ use should be viewed as an attempt to deal with anxiety or looms as a form of addiction, is a dubious question. Lately, withdrawal from BDZ has been regarded with increasing concern, and cases of symptomatic withdrawal have been documented for dosages even lower than those taken on average by MMT patients [83]. BDZ-abusing MMT patients may display oversleeping, ataxia, speech difficulties, and even anger attacks [74]. Diazepam addiction has partly replaced the known phenomenon of dependence on hypnotics, which are often carelessly prescribed for insomnia by GPs. Diazepam use, in SUD patients, can sometimes produce dreamlike states of consciousness, which SUD patients may experience as optimum conditions for getting involved in illegal behaviors.

In those circumstances, terrible accidents may happen, so the prescription of BDZs to SUD patients should only be allowed when strictly necessary. HUD patients should never be given free access to BDZ medications. Specifically, it is harmful to encourage SUD patients to decrease their methadone dosage and use BDZs to compensate for the difference: Not only will patients’ clinical conditions not improve, but they will also be put at risk of developing a poly-addictive disease [84].

It is not a question of what the dynamics may be that underlie BDZ use. It can certainly be expected to worsen an addict’s already delicate conditions, especially if the patient has been using BDZs heavily upon entry. Finances and time did not allow for investigation of possible contributions from non-medical interventions provided in the program. In general, these interventions can be classified as self-regulation training; Cognitive Behavioural Therapy (CBT ) for thought stopping and positive self-talk; EMDR for trauma processing; energy psychology and ear acupuncture for urge reduction; and dialectical behavior therapy groups for developing social skills and healthy habits. Future plans will include outcome studies of these and related interventions.

The use of BDZ by patients on MMT has the effect of complicating the clinical picture. The relative safety of BDZ use by methadone-treated patients has still not been examined systematically. It is not yet clear whether a maintenance strategy with clonazepam is a useful BZD treatment modality for BZD-dependent MMT patients with a long-term history of substance use and previous attempts at detoxification. In the case of our patients, who were treated with clonazepam maintenance during MMT, we have collected detailed information regarding their outcomes. In our sample treated with a methadone-clonazepam combination, the retention rate, at eight years, was 57.1%. Baseline-endpoint improvements were significant for global clinical impression and the level of social adjustment. Patients with a severe comorbid dependence, when treated with over-standard dosages of methadone and co-treated with (clonazepam maintenance treatment) CMT, may have outcomes that are satisfactory as long as they are maintained on their medication in the long term. According to the experience of our V.P. Dole Research Group, it is possible to combine up to 380 mg/daily of methadone and 30 mg/daily of Sodium gamma-hydroxybutyrate (GHB) [57,85].

In a naturalistic (observational) controlled cohort study [86] we compared the long-term outcomes of treatment-resistant HUD patients with (HA+BDZ) and without (HA-BDZ) severe comorbid BDZ addiction. 63 HA-BDZ and 14 HA+BDZ patients were monitored prospectively along an enhanced MMT program (MMTP). HA+BDZ patients were, in addition, treated, with CMT. Survival-in-treatment rates were no different in HA+BDZ and HA-BDZ patients. HA+BDZ patients showed better outcome results than HA-BDZ patients. HA+BDZ patients needed a higher methadone dosage in the stabilization phase. 

These studies support the possibility of using MMT and clonazepam maintenance combination in heroin-addicted patients with comorbid severe BDZ dependence. At this point, our V.P. Dole DD-RG further proposed the use of clonazepam also in the stabilization and long-term maintenance of all those suffering from strong BDZ dependence [87].

The findings emerging from our V.P. Dole DD-RG experience indicate that the average methadone dosage needed to stabilize HUD patients with a DD of anxiety disorder is lower (80 mg/day) than the average required to maintain other types of DD/HUD patients, or even uncomplicated patients (100 mg/day). The combination with standard doses of serotoninergic and tricyclic medications is possible, but requires caution during the induction phase of MMT or if the patient is already in treatment [57]. In our studies, consistently with such observations, naltrexone has been shown to elicit anxiety in non-SUD, as well as HUD patients [59]. The anxiety disorders of HUD patients can also be treated successfully with antidepressant drugs and buspirone [88]. SSRIs, tricyclic agents, and trazodone are effective in controlling both anxiety and depressive symptoms and are suitable for long-term treatment programs. Imipramine and nortriptyline may cause sedation and hypotension.

## 5. Treatment of Psychotic Disorders during MMT 

### 5.1. Psychosis and SUDs

Previous suggestions [89] about a possible causal relationship between chronic morphine use and the onset of a psychotic picture failed to find subsequent confirmation [90,91]. Data on the comorbidity of SUD strengthen the assessment that the likelihood of a psychotic spectrum diagnosis among HUD patients on MMT is low. In the Yale study, only about 3% of patients were diagnosed as affected by schizophrenia (0.2%) or schizoaffective disorder (3.2%), so promoting doubts about the reliability of formerly reported prevalence rates [92], which showed an approximate range between 10–20% in different surveys [93,94]. Moreover, the significant studies [95,96,97,98] that have investigated the prevalence of SUD in populations of schizophrenics have reported heroin use as being found in around 2–7% of subjects, a range that decreases below its prevalence among the USA general population, which showed an approximate peak as high as 9% in an old NIDA survey [99]. The prevalence of amphetamine and hallucinogen use is more significant among schizophrenic subjects than in the general population—25% vs. 15% and 20% vs. 15%, respectively [95,100].

Some authors [97,100] speculate that chronic psychotic patients self-select pro-dopaminergic substances, on the expectation that they will be effective in mitigating their negative symptoms, comprising natural or iatrogenic depression and extrapyramidal effects, due to neuroleptic medications. The dopamine-wasting effect of any psychostimulant drug may itself lead to the persistence of addictive behaviors, through the attempt to maintain a normal dopaminergic firing level. This mechanism is similar to cocaine-induced dopaminergic stress, through which a dopaminergic hypofunction perpetuates the tendency to resort to cocaine.

Regarding other non-therapeutic substances, we can consider a number of different points [101]. Mescaline, psilocybin and LSD are straightforward psychotomimetic and hallucinogenic substances, because they can cause psychopathological syndromes displaying the same features as those of non-SUD psychotic disorders. Cocaine and amphetamines and, to a lesser extent, cannabinoids may produce a range of thought or sensorial-perceptive alterations which can reach the same degree of severity as full-blown psychotic states [102,103,104,105,106,107,108]. These effects are entirely dependent on the specific action of these drugs on the dopaminergic system, which is hyperactive in the brain of acutely psychotic patients. Acute substance-induced psychosis is generally of short duration, most usually between days and weeks. It is common, however, to witness the persistence of psychotic symptoms, along with the course of a schizophrenic-like prognosis. Different interpretations of such pictures are possible. They might occur in individuals who use drugs as a result of their previous psychopathological condition. They might also apply to persons who are prone to unpredictable psychotic-like experiences as a function of a specific excitatory effect of substances—an effect shared with stressful events. Lastly, the substance could be directly and individually responsible for the onset of a psychotic picture in low-risk populations.

Acute psychosis can be documented in chronic cocaine users without previous psychiatric comorbidity after an average of three years of continuous use. Such events usually achieve resolution spontaneously as long as cocaine use ceases, and is not prolonged after the so-called crash phase, which is characterized by psychomotor retardation and oversleeping. The chronic intoxication caused by cocaine or amphetamines has also been associated with chronic psychoses, which continue independently, displaying chronic psychotic symptoms without co-occurring cognitive failures. Liability to the development of chronic psychosis does not differ with the pattern of cocaine use. The factors associated with the premorbid personality are therefore likely to be involved [109,110,111].

### 5.2. Antipsychotic Medications

Both typical and atypical antipsychotics have been tested in DD/HUD psychotic patients. We are convinced that any evaluation of antipsychotic medications must take into account the impact on drug-related issues: Substances used may have psychotomimetic properties, and the persistence of, or relapses into, drug-taking are both predictive of an unfavorable course.

Typical antipsychotics (TAs) offer little help to DD psychotics [112,113,114,115,116,117]. Substance misuse is common among people with schizophrenia treated with TAs, and it shows no reduction during treatment; a tendency towards an increase in consumption during treatment has emerged for some substances, such as nicotine [118,119]. Psychotic SUD patients show a less favorable response to TAs, presumably due to the pro-psychotic effects of frequently used substances, which reduce the incisiveness of that treatment. When substance use foreruns a psychotic episode, agents such as haloperidol or perphenazine may prove to be less effective than expected.

Both TAs and SUD act on the CNS dopaminergic system, so it can be hypothesized that particular phenomena may be related to the pharmacodynamics of the specific agent and its impact on the course of psychoses [112,115]. At clinically effective dosages, it has been demonstrated that TAs put off the mesolimbic dopaminergic firing, which is the known substrate for the rewarding effects of many used substances, including cocaine. Alcohol and cocaine are the two most frequently used drugs among psychotic patients. The majority of addictive substances induces increased levels of omovanillic acid (OVA), an index of dopaminergic activity, and heighten the release of dopamine in the nucleus accumbens, which is the terminal of the dopaminergic mesolimbic pathway [120]. On this basis, we can say that substance use is effective in reversing the dopaminergic blockade induced by TAs.

This fact is consistent with the relapse-provoking role of substance use, and it suggests that treated psychotic patients may resort to substances to counteract the blunting effect on emotional life brought about by the mesolimbic antagonism of TAs. In a highly sophisticated mesolimbic system, like that of users, which is more sensitive to lack of stimulation than that of healthy individuals, the administration of TAs is likely to bring out an intense and painful hypophoria, followed by compensatory behavioral activation towards rewarding sources. Relapsing into the resumption of the use of available substances would automatically ensure compensation in individuals who have already learned to achieve rewards by substance use before treatment. The use-enhancing effect of TAs would be positively correlated with the antidopaminergic potency of the specific medication. Consistently with that, the add-on of desipramine in cocaine-using psychotic patients has been reported to reduce cocaine use, which does not happen among non-psychotic cocaine users. In other words, TAs appear to increase drug use in a way that is reversible by desipramine, which has a useful impact on drug use to the extent to which it counteracts the mesolimbic dopaminergic antagonism expressed by TA.

When compared with TAs, clozapine, which possesses low specificity on dopaminergic receptors, showed less capacity to reduce dopaminergic transmission in animal models. So too, in animal models, clozapine, unlike other antipsychotics, has been shown to reduce cocaine use, at fixed doses, and to lengthen cocaine-free periods, when an increased dosage is used. On clinical grounds, clozapine has revealed anti-craving properties. Firstly, the efficacy of clozapine is not related to patient concurrent substance use, in a way not attainable with TAs, which, as a rule, prove to be less effective in SUD patients. Some authors as also our research group have even suggested that SUD psychotic patients may display a better response to clozapine than non-users [121,122,123,124].

In DD schizophrenics, clozapine treatment reduces nicotine use. Passing from haloperidol to clozapine reduced nicotine use, whereas haloperidol had caused an increasing use. The clozapine-related decrement in nicotine use is dose-related [119]. AUD patients treated with clozapine are likely to have stayed abstinent (50%) throughout the first year after discharge from the hospital. Two psychotic patients with AUD, after treatment with 500 mg/day clozapine, were shown to have stayed abstinent in the long term [125,126].

The interpretation of clozapine’s effects on drug and alcohol use is not clear. In some contexts, an anti-craving primary impact seems to be present, whereas in others it looks possible that drug use leads to a reduction, because in its case there is no need for self-medication brought about by an antidopaminergic blockade, such as that which has to be dealt with in the case of TAs [127,128]. Abusing schizophrenics tend to show ‘negative symptoms’, anxiety and mood especially, to a lesser extent, whereas counterbalance by dopaminergic substances ends up by exacerbating psychotic symptoms, so unfavorably affecting the natural course of the illness, and reducing the efficacy of antidopaminergic antipsychotics (i.e., TAs). Negative symptoms, treatment by TAs, the use of dopaminergic substances, psychotic relapses, and then the potentiation of TA treatment to achieve a broader antipsychotic defense spectrum, end up forming a vicious circle.

In DD patients, TA-induced hypophoria could be the key to a clarification of the dynamics between antipsychotic treatment and the natural course of a co-occurrent SUD. The frequency of depressed mood among TA-treated psychotics and its partial reversal following drug-taking are consistent with this explanatory model. The novelty-seeking dimension of Cloninger’s Tridimensional Personality Questionnaire (TPQ), which implies higher drug-seeking risk, has been associated with the D4 receptor subtype. Antagonist D4 medications may reduce drug-seeking behavior, whereas D2 antagonists (such as TAs) appear to increase them, especially in individuals who are highly favorable to D4. In reality, clozapine’s profile is distinguished by its higher specificity for D4 receptors (higher D4/D2 ratio) [129]. Risperidone, which shows the highest specificity for D4 receptors, has not yet been wholly evaluated on this issue.

### 5.3. Methadone and Antipsychotic Medications

The concurrent use of antipsychotics in MM-HUD psychotic patients can be considered acceptable and helpful [130,131]. Low dosages of TAs, such as chlorpromazine, fluphenazine and haloperidol can control psychotic symptoms, when combined with methadone [25,132]. Antipsychotics are quite likely to be poorly tolerated by HUD patients. Generally speaking, TAs are not used, but, if they are, patients should be urged to comply. Depot preparations make it possible to resolve the patient’s non-compliance, and concurrent MMT seems to act as a shield against extrapyramidal side-effects.

Standard stabilization doses of methadone and antipsychotic medications can be used in treating DD psychotic patients [57]. During the induction phase, clinicians should be particularly careful, to minimize the narcotic co-potentiation of antipsychotics and opiates, notably when TAs are used. Our recommendation is to avoid administering antipsychotics until the steady state has been reached with methadone. During this period, the sedative action of methadone itself can be utilized. Besides, the use of BDZs cannot be recommended. When psychomotor excitement is severe and requires antipsychotics administration, a limited number of antipsychotics can be used, always under medical supervision, and paying attention to ensure that antipsychotic doses are not taken late in the evening. Central antihistaminic medications are a valid and suitable alternative option in aiming to achieve sedation in psychotic HUD patients.

Over-standard methadone doses are in general needed in the treatment of DD-HUD patients displaying concomitant high-severity psychopathological symptomatology [133]. In one of our studies, we compared the long-term outcomes between treatment-resistant psychotic HUD patients (PSY-HUD) and peers without DD (HUD). Eighty-five treatment-resistant HUD patients—25 of them affected by chronic psychosis and 60 without DD—were monitored prospectively for up to eight years while continuing to receive enhanced MMT. Continuation in the treatment of PSY-HUD patients was 36%, compared with 34% for HUD patients (*p* = 0.872). After three years of therapy, these rates tended to become progressively more stable.

Regarding CGI severity and DSM-IV-GAF, PSY-HUD patients showed better outcomes than HUD patients. No significant differences were found regarding positive toxicological results or the methadone dosages used to achieve stabilization. The time required to stabilize PSY-HUD patients was shorter (*p* = 0.034). An enhanced MM treatment seems to be equally effective in patients with PSY-HUD and those with HUD [134].

### 5.4. Disulfiram

Disulfiram works in limiting alcohol consumption independently of the presence of psychotic symptoms. The decrease in alcohol use is bound to have a positive impact on the natural history of psychosis itself. Alcohol is known to worsen psychotic symptoms. In patients treated with high-dose disulfiram, however, psychotic symptoms have been reported to deteriorate [129,135]. Schizophrenic AUD patients have been reported to benefit from disulfiram treatment to the same extent as non-psychotic AUD patients. In particular, alcohol use in people with schizophrenia seems to show an excellent response to the clozapine-disulfiram combination [135]. Disulfiram is useful in psychotic AUD patients at a dosage of 250 mg/day. At this dose, the likelihood of an iatrogenic worsening of psychotic effects carries less weight than the impact of ongoing alcohol use in developing symptomatology and in harming the overall course of the illness. Disulfiram is also useful in treating cocaine dependence in methadone-maintained HUD disorders [136].

### 5.5. Desipramine

Desipramine has been used at doses of 100–150 mg/day in cocaine-addicted psychotics, as the add-on to antipsychotic treatment. That combination reduced cocaine craving considerably. Desipramine, when tried on non-psychotic cocaine-SUD patients, failed to show any specific efficacy [137,138]. Anti-craving dopaminergic medications must be avoided during acute psychotic phases, enhancing the risk of exacerbating psychotic symptoms, and have an uncertainty impact on substance use. In stabilized MM HUD psychotic patients, our anecdotal evidence suggests that ropinirole, up to 1.5 mg/day, can reduce the craving, with no concurrent psychopathological destabilization.

### 5.6. Proposals

We propose the following strategies when treating DD patients with psychotic disorders:

- Antipsychotic properties of long-acting opiates can be applied.
- Patients’ greater compliance during MMT can be used to reduce the risk of psychosis crises.
- Low doses of typical or atypical neuroleptics (combined with mood stabilizers) can be added on, so taking advantage of methadone and antipsychotic blood level increases.
- Clozapine-like antipsychotic medications must be preferred.
- If a withdrawal psychosis is present, methadone must be reintroduced.
- Antipsychotic medications must be used with caution in low tolerance psychotic MM HUD patients, especially during the MMT induction phase.
- In MM HUD patients, low potency antipsychotic medications must be avoided because a higher dose means greater metabolic interference that produces greater blood level increases in opioids.
- For agitated psychotic MM HUD patients, intramuscularly central antihistaminic medications must be preferred

## 6. Treatment of Violence during Methadone Maintenance Treatment

The assessment of the role of opioids in modulating aggressive behavior is no easy matter, as most studies on the subject deal with animal models, where acts of aggression result in defensive behavior against preying. In the literature a variety of evidence allows the following conclusions to be reached [139,140,141,142,143,144].

Several brain areas that are related to the production and modulation of defensive behavior are crowded with opioid receptors and enkephalin-binding axon terminals. These areas comprise:

- The “nucleus accumbens” and the “nucleus proprius” of the “terminal stria” [145,146,147,148,149,150,151].
- The periaqueductal grey substance, which produces/enhances defense [152,153,154].

The administration of naloxone heightens or elicits protective behavior and aggression. Naltrexone was unsuccessful when used to modulate defense in monkeys, while in mice it caused more frequent aggressive attacks. Most of the evidence suggests that the role of opioid modulation differs from the typology of aggression [155,156,157,158,159,160,161,162].

Naloxone-challenged cats showed greater aptitude to defensive behaviors, regarding a shortened latency of reaction and a lowered threshold. The measured effects depended on time and dosage. In the same model, preying behaviors showed that cats had acquired a more extended period of latency after naloxone administration [141].

In a review of our V.P. Dole DD-RG [163] we discussed the correlations between aggressiveness, defined according to a behaviorist model, and heroin dependence according to DSM criteria. Criminality appears to be only an indirect, partial index of aggressive behavior in HUD patients. The violent behavior of HUD patients is probably different from that of other kinds of mentally ill patients, non-opiate substance users and the general population, and seems to be specifically related to the degree of chronic intoxication. Gender differences, violent habits before heroin use, and modulation during intoxication or withdrawal states have been documented. The association between cerebral opioidergic abnormalities and psychiatric disorders characterized by affective instability, feelings of anger and hostility, perception abnormalities and sexual dysfunction, could explain highly aggressive behaviors of HUD patients which are not directly related to drug supply. Knowledge about the anti-aggressive property of non-opioid drugs is limited. On the other hand, opioid agonists are promising agents for the treatment of aggressive behaviors in non-addicted patients, too.

In another of our studies, we demonstrated the following characteristics of violent HUD patients entering AOT [164]. We evaluated the violent behavior of 252 heroin-dependent patients (163 males and 89 females) at treatment entry, and we compared them with the general Italian population (standardization sample). We also evaluated correlations between violent behavior and the addiction history of our patients. We recorded the nature of violent reaction by applying the Buss-Durke Inventory (BDI) and patients’ addiction history through our Drug Addiction History Questionnaire. Overall, heroin-dependent patients obtained higher scores than the general population. Specifically, the top values were concentrated in the suspicion, resentment and assault dimensions whereas the lowest ones centered on irritability and verbal aggression. Feelings of guilt were more severe than in general populations. Only about 20% showed a weak aggression profile; 3 out of every four patients were characterized by violence arising from to suspicion and resentment (type 2 according to our V.P. Dole DD-RG). Compared with the general population, a higher number of heroin-dependent patients showed an aggressive type 2 profile. Addiction history and type and degree of aggressive behavior showed a non-robust correlation, only found with periods of voluntary or forced abstinence, legal problems, altered mental status, and social leisure activity). In conclusion, at treatment entry, HUD patients showed more violence than the general population; that violence was related to an altered mental state and was only weakly correlated with addiction history.

### 6.1. Opiates as Anti-Aggressive Medications

When intervening with SUD patients, the top priority is to control possibly homicidal or suicidal patients and metabolically impaired ones. In the first two cases, hospitalization is necessary; whereas in the third, outpatient treatment is sometimes enough.

On clinical grounds, antidepressant medications do buffer the risk of suicide in addicted patients. In the experience of our V.P. Dole DD-RG, this risk appeared to be higher among naltrexone-treated patients, and lower in methadone-maintained ones. A series of studies indicates that agonist opiates can be useful in controlling concurrent psychopathology and aggression in HUD patients. In one of our studies, we examined over 600 street HUD patients who asked for treatment. 30% of these, reported suicidal ideation, but a high degree of severity was only highlighted in 1% of cases. Hostility and anger were found in as many as 40% but were displayed in a severe form in only 4%. Violent behavior occurs most often among non-depressed and phobic HUD subjects. Suicidal thoughts and violent action are quite common among street SUD patients applying for treatment at our clinic; our view is that these subjects may have such a profoundly impaired opioid function that it can no longer be alleviated even by the highest heroin street doses. In our personal experience, most HUD patients search for treatment when they cannot find enough money to ensure their daily heroin supply. We suppose that violent behavior depends on undermedication, consistently with the fact that subjects were displaying more severe psychopathology, such as depression, anxiety, paranoia, somatic symptoms and aggression, at treatment entrance, and need higher stabilization dosages [165].

In particular, we found an inverse correlation between violent behavior and methadone dosage. We also demonstrated that DD/HUD patients need higher stabilization dosages (150 mg/day on average) than HUD patients without psychiatric comorbidity (whose average dose is 100 mg/day). When adequate dosages are used, retention rates do not vary with the presence or absence of DD [133,166]. There is a trend towards lower treatment retention for DD/HUD patients during the early period of care, but this trend seems to show a crossover pattern after the first three years. DD SUD patients are more likely to stay in treatment after three years. Bipolar 1 patients are an exception to this rule, as they continue to show a lower retention rate [52].

More information about the relationship between opiates and aggression comes from our clinical experience on agonist- or antagonist-maintained HUD patients [62]. When SUD patients were compared regarding violent behavior during a monthly follow-up, significant differences were observed between methadone and naltrexone-maintained patients. Methadone-maintained patients displayed lower levels of aggression and self-injuring behavior. Patients did not differ at the beginning of treatment, but methadone-maintained patients were less aggressive at the end of the observation period. The undesirable effects of naltrexone in controlling aggressive behavior were also documented in a sample of bulimic patients, who had been treated with naltrexone alone or naltrexone plus fluoxetine, in a three-month monthly crossover protocol [167]. In the same study, we described a bulimic patient who had developed panic attacks in the early phase of treatment with naltrexone [59]. Naltrexone is likely to be responsible for the discomfort observed in naltrexone-maintained patients; the addition of fluoxetine to naltrexone improved the retention rate of naltrexone-maintained subjects. We have suggested that fluoxetine is useful in overcoming some of the naltrexone-induced resistance to retention in naltrexone treatment [64].

According to our research results, the opioid system may be closely involved in the control of aggressive behavior. Undeniably, when SUD patients who take enough heroin are given enough agonist to balance their opioid tolerance, they do not display violent or suicidal behaviors. Violence, whether as self-injuring behavior or as outward violence, only characterizes HUD patients whose tolerance to opioids has become unbalanced by a high level of opioid incentive. Among non-SUD subjects, violent or suicidal individuals may be marked by a primary imbalance of their endogenous opioid system. According to this hypothesis, a higher level of endorphins was documented in autistic subjects, who had become unbalanced by their corresponding tolerance towards opiates [168]. The administration of an antagonist of opioids to autistics was not followed by any withdrawal symptoms, as in SUD patients [169,170]. Violent subjects may constantly display a reduced functioning of their opioid system, comparable with what HUD patients end up by suffering from, due to long-term exposure to toxic opiates. On clinical grounds, the violent behavior of HUD patients mostly looms as a sign of metabolic impairment. Violent HUD patients require higher methadone doses than their non-aggressive peers. If violence appears during agonist-treatment, an increased dosage is probably needed.

SUD patients have been generally considered as essentially psychopaths—violent individuals who unconsciously desire death. This view appears to be incorrect: Violence can best be considered as a sign of SUD and deserves more appropriate medical intervention than social stigma and stricter repression.

As a fall in levels of violence follows adequate MMT, it can be hypothesized that some relapsing into heroin usage by HUD patients works as a means of self-medication, rather than as a way to seek euphoria. According to Khantzian [127], aggressive symptoms are among the features that may occur among the habits of self-medication.

Opiate agonists display an anti-aggressive action both against self-injuring behavior and against outward violence. This fact is interesting because of the lack of anti-aggressive medicines, on the one hand, and the frequency of violent syndromes among psychiatric patients, on the other. Apart from clozapine [171,172], in fact, antipsychotic agents show a reduced ability to control violence outside a psychotic condition. According to Khantzian, we may state that in normal circumstances, and during development, the brain produces endorphins not only to limit pain, but also to maintain affective balance and well-being. Endogenous opioids may be central to the modulation of human aggression, which is essential to survival, but is also devastating when it becomes over-controlled. By studying the endorphins in mental activities, a better understanding can be achieved of how to increase energy and activity without eliciting violence, and about how the dysfunction and abnormality of the opioid system may be related to the negative character of human violence [127].

## 7. Treatment of Alcohol Use Disorder (AUD) during MMT

In the literature, the relationship between depressive states and alcohol use, as an issue still at the center of sharp controversy, continues to attract attention to the dynamics that link different kinds of depressive states and alcohol-related problems. The majority of authors agree in considering heavy drinking as an equivalent, or a masked form, of depression [173]. Drinking patients, despite severe or advanced somatic consequences, display a peculiar form of depression [173]. AUD is characterized by depressive states, which, however, are mostly of minor severity and take a disguised form [174]. Other authors have described a strong association between bipolar disorders and AUD. According to Kraepelin, almost 25% of bipolar patients had a clear preference for alcohol [175].

Several authors conclude that alcohol use mostly characterizes depressive states and is a way to elate mood and soothe pain, so alcohol use during states of mood elation is a sign of excitement and impulsiveness [176]. Also, our studies have suggested a close link between cyclothymia and alcohol use [177,178]. Chronic depression too has been associated with AUD. Not surprisingly, alcohol use can stand as an addictive disease itself in some cases, and it is often found co-present with SUD in general. In the literature we can find many studies increasingly reporting an association between heroin and alcohol use [17,179,180,181,182,183,184,185,186,187,188,189]. Alcohol use seems to be related to polydrug use, and mainly affects young SUD patients. In this population, lifetime rates for AUD range between 10 and 75%. The National Drug Alcohol Collaborative Project (NDACP) found a rate of about 40% for combined AUD/HUD in a sample of over 1,500 HUD patients [182]. Heroin was the first substance of use in 99% of cases. Rounsaville depicted a lifetime and index prevalence of AUD of about 15% and 35%, respectively [190]. Californian HUD patients have been reported to use alcohol at a rate of about 50–75%, and about 10% have been admitted to hospitals for alcohol-related physical matters. AUD occurs as often as 10–20% among street SUD patients, and up to approximately 30% among methadone-maintained subjects [17,184]. Some authors studied the increase in alcohol use during MMT programs, concluding that methadone-maintained HUD patients may use alcohol to counter the opioid-normalizing effect of methadone, and to go beyond the methadone-heightened opioid threshold [17,184,191]. When the correlation between alcohol and heroin use among MMT HUD patients was examined in a large sample of HUD patients, it was pointed out that alcohol use during MMT seems to be the expression of an automatic behavioral pattern, according to which alcohol use tends to grow as street-opiate use falls, and the reverse [17]. Additionally, Rounsaville, who supports this theory, also reports that AUD is mostly found in SUD patients who had once misused alcohol, so displaying a relapse into a previous alcohol-related disorder [190].

On the basis of their clinical experience, Maremmani and Shinderman suggest that the use of alcohol, BDZs and other types of drug in HUD patients may be correlated with a condition of opiate tolerance not compensated by street opioids or by agonist opioid medication doses. Thus, the use of an appropriate methadone dosage during MMT is vital not only, because it increments the retention rate for patients within the treatment group, so allowing an improvement in patients’ social adjustment, but also because it reduces the risk of polydrug use [23,84].

### 7.1. Psychopharmacotherapy of AUD-HUD Patients

Undoubtedly, alcohol has a negative influence on the outcome of an MMT. It implies a more severe behavioral and cognitive disturbance, a higher prevalence of psychiatric disorders, and lower compliance, which often conditions a treatment provider towards a premature exit of the patients from treatment [96,192]. Moreover, AUD has more physically severe consequences, such as chronic hepatic failure. It can lead to premature death or may favor overdosing accidents, due to interference with the methadone metabolism [193]. Since both addictive disorders need to be treated at the same time, disulfiram was tried first on methadone-maintained patients, but, though the complete safety of the combination was ascertained [194,195,196], its efficacy is still controversial, as disulfiram is mostly equivalent to placebo [195]. The decrease in alcohol intake appears to depend on a subject’s compliance with the combined treatment and also on the level of the subject’s awareness of the severity of the problem [195]. It is awkward to invite AUD patients to take disulfiram daily. Subcutaneous implantations can be resorted to, where patients give their consent and MMT may be allowed if patients continue to show compliance with disulfiram treatment. Another strategy is not to treat patients with methadone if there is a positive result to the screening test for alcohol use during the previous 12 hours or abnormally high alcohol blood levels have been found. However, this procedure does not guarantee that patients will abstain from alcohol after their methadone has been administered. According to the experience of our V.P. Dole DD-RG, it is possible to combine up to 380 mg/daily of methadone with 30 mg/daily of GHB [57].

The combination of methadone and disulfiram should be limited to the most severe cases, or, at least, to cases in which non-compliance has limited another treatment possibility. In all other cases, supportive approaches, psychosocial treatment and different pharmacotherapies should be used. Naltrexone, while useful in pure AUD, is unsuitable for AUD HUD patients. During naltrexone treatment, substance use (for instance, of BDZs and stimulants) has been reported to increase [21]. One possible explanation is the following: Heroin can induce an intense craving, which reinforces the need to search for heroin and heroin intake. Naltrexone blocks the opioid-induced reward, so leading reward craving to extinction, but at the same time, it intensifies the hypophoria caused by the impossibility of an opioid stimulation. Naltrexone-treated patients may, therefore, resort to alcohol or BDZ to cope with late withdrawal symptoms and naltrexone-enhanced hypophoria.

### 7.2. GHB for AUD-HUD Patients

The GHB is an old general anesthetic medication that is no longer used for its original purpose. GHB shows several pharmacological properties. At anesthetic dosages, it causes an increase in dopamine levels in several cerebral areas, which follows a pervasive inhibition of CNS neuronal activity. At lower dosages it seems to selectively raise dopamine transmission in the mesencephalic ventral tegmental area [197,198,199,200,201]. Some of GHB’s pharmacological properties are of particular interest: It binds none of the sites associated with GABA-A receptors, but it does bind to GABA-B receptors; it is a substitute for ethanol in rats, and it has been proved to decrease ethanol consumption in AUD patients [202,203,204,205]. GHB may be used in AUD/HUD patients, and be added, at high dosages, to MMT, which can control heroin use with its blocking dosages [206].

We can report the case of a female AUD/HUD patient, who became stabilized on MMT at our outpatient clinic. F.M. was a 31-year-old woman, with a 10-year history of HUD, poly-user and HIV-positive. After treatment with 10 mg/day methadone at a Local Addiction Unit, she developed an AUD. She was judged to be one of the most severe cases ever seen by us. After one month of treatment, she had limited her alcohol consumption by 70%, and the global clinical judgement indicated a mild form of the disease, so recording a major therapeutic gain combined with the absence of significant side-effects. She was treated with GHB at an average dose of 27 ccs/day (the range was 20–30), adding 27 mg/day average dose of methadone (with a range of 10–30) and clonazepam, on average 4.75 mg/day (min. 2, max. 9). Trimipramine, 100 mg, was added on in the evening to control insomnia. During the subsequent phases of stabilization and maintenance, GHB dose was gradually increased up to 60 ccs/day, to be maintained for at least one year. MMT lasted seven years until the patient died due to AIDS while still receiving still 40 mg/day of methadone, while GHB, previously given at ten ccs/day, had been tapered off.

According to our clinical experience [206] GHB is one of the most effective options available for the treatment of hard-core AUD in maintenance programs that aim to achieve relapse prevention and rehabilitation [207]. Polysubstance use and multiple addictions have become quite common in alcoholic youths and former HUD patients receiving inadequate or no specific treatment. In approaching these categories, GHB is usually neglected, as a result of the idea that its use potential must be amplified in use-prone individuals. However, the normalizing effects of anti-craving treatment on the behavior of HUD patients may make GHB a suitable remedy for the heroin-alcohol polyuse picture. The same cannot be said of cocaine users, due to the lack of anti-craving treatments possessing significant, reliable effectiveness. We have described 13 cases of AUD HUD patients, in which GHB proved to maintain some efficiency, even if there were substantial limitations regarding compliance and completeness of response.

## 8. Final Remarks

Our main aspiration includes increasing the accessibility to treatment, raising HUD patients’ compliance and taking a medical approach to the SUD. Achieving rapid, complete control of acute phases is imperative. Long term treatment becomes possible if the patient can be detoxified or if MMT can be initiated in line with the patient’s opiate tolerance. After this phase, it is necessary to stabilize residual symptoms (in the subacute phase) and maintain good outcomes in the long term (case management). It is generally possible to detoxify patients from psychostimulants, hallucinogenic drugs or cannabinoids before starting any psychopharmacological medications, but, if concomitant HUD is present, patients must be treated with MMT. The prescription of BDZs, which are abusable psychotropics must be assessed with great caution. For polyuser HUD patients, it is reasonable to detoxify them from different substances one by one, during MMT.

Doctors, who are responsible for treating DD patients must often have to cope with some misconceptions. The first is that DD/HUD patients are unresponsive to standard treatments for HUD. The second is that they are, on the whole, non-compliant with the treatment. The third is that they will show a less satisfactory outcome.

During our clinical experience over many years, we have observed that the stay-in-treatment of our patients is significantly higher among DD/HUD patients than among solely HUD patients [133,134].

In conclusion, we can state that DD-SUD patients should, in all cases, be treated for their SUD by using blocking dosages of methadone, which can be expected to be higher than those required to treat uncomplicated HUD patients while considering stabilization as a medium-term goal. As noted, treatment benefits for some DD/HUD patients may not only reduce the use of addictive substances, but also ameliorate certain psychological symptoms. In this context, opioid agonist medications should be considered for their possible efficacy in reducing craving and also in having a beneficial psychotropic effect for persons with depressive, anxious, and psychotic symptoms.
